# Supplementary figures and images for: Analytical Separation of Closantel Enantiomers by HPLC
Source: Molecules. 2021 Nov 30;26(23):7288. doi: 10.3390/molecules26237288 (PMC8659055; doi:10.3390/molecules26237288)

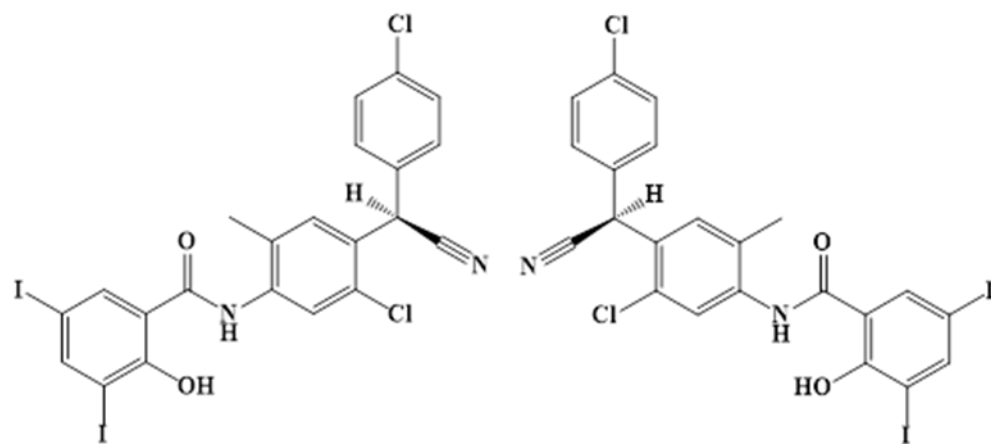

**Figure S1.** The structure of closantel enantiomers

Supplement: Supplementary file 1 [file molecules-26-07288-s001.zip › molecules-1426177-supplementary.pdf]
